# Work environment and safety climate in the Swedish merchant fleet

**DOI:** 10.1007/s00420-016-1180-0

**Published:** 2016-11-04

**Authors:** Karl Forsell, Helena Eriksson, Bengt Järvholm, Monica Lundh, Eva Andersson, Ralph Nilsson

**Affiliations:** 1 Occupational and Environmental Medicine, University of Gothenburg, Gothenburg, Sweden; 2 Occupational and Environmental Medicine, University of Umeå, Umeå, Sweden; 3 Shipping and Marine Technology, Chalmers University of Technology, Gothenburg, Sweden; 4 Occupational and Environmental Medicine, Norrlands Universitetssjukhus, 901 85 Umeå, Sweden

**Keywords:** Web-based survey, Seafarers, Work environment, Safety

## Abstract

**Purpose:**

To get knowledge of the work environment for seafarers sailing under the Swedish flag, in terms of safety climate, ergonomical, chemical and psychosocial exposures, and the seafarers self-rated health and work ability.

**Methods:**

A Web-based questionnaire was sent to all seafarers with a personal e-mail address in the Swedish Maritime Registry (*N* = 5608). Comparisons were made mainly within the study population, using Student’s *t* test, prevalence odds ratios and logistic regressions with 95% confidence intervals.

**Results:**

The response rate was 35% (*N* = 1972; 10% women, 90% men), with 61% of the respondents working on deck, 31% in the engine room and 7% in the catering/service department (1% not classifiable). Strain on neck, arm or back and heavy lifting were associated with female gender (*p* = 0.0001) and younger age (below or above 30 years of age, *p* < 0.0001). Exposures to exhausts, oils and dust were commonly reported. Major work problems were noise, risk of an accident and vibrations from the hull of the ship. The safety climate was high in comparison with that in land-based occupations. One-fourth had experienced personal harassment or bullying during last year of service.

**Conclusions:**

Noise, risk of accidents, hand/arm and whole-body vibrations and psychosocial factors such as harassment were commonly reported work environment problems among seafarers within the Swedish merchant fleet.

**Electronic supplementary material:**

The online version of this article (doi:10.1007/s00420-016-1180-0) contains supplementary material, which is available to authorized users.

## Introduction

Several studies have shown higher mortality and morbidity ratios in seafarers when compared to land-based occupations. Recently, Borch et al. (2012) showed a sixfold elevated mortality in Danish seafarers due to accidents on board, including ship wrecking. Cancer is more common in seafarers, with especially lung cancer and mesothelioma being more common in engine room crew and lymphohematopoietic cancers in tanker crews (Bianchi et al. 2005; Kaerlev et al. 2005; Moen et al. 1990, 1994; Nilsson 1998; Nilsson et al. 1998; Peto et al. 1999; Pukkala et al. 2009; Pukkala and Saarni 1996; Rafnsson and Gunnarsdottir 1995; Rafnsson and Sulem 2003; Saarni et al. 2002; Sulem and Rafnsson 2003). Ischemic heart disease and psychiatric diagnoses, including suicides, have also been reported to be more common among seafarers (Bloor 2000; Brandt et al. 1994; Elo 1985; Hemmingsson et al. 1997; Jensen 1996; Moen et al. 1994; Nilsson 1998; Nilsson et al. 1998; Pukkala et al. 2009; Pukkala and Saarni 1996; Rafnsson and Gunnarsdottir 1994, 1995; Rafnsson and Sulem 2003; Saarni et al. 2002).

Work exposures that might be relevant for cancer development are exposures to asbestos, polycyclic aromatic hydrocarbons (PAH) and nitroarenes, soot and oils, especially for engine room crew members, benzene, for deck crew members on board tankers, and diesel engine exhausts, for example when working on deck during loading and unloading of vehicles (Attfield et al. 2012; Boffetta et al. 2001; Bruske-Hohlfeld et al. 1999; Jarvholm and Silverman 2003; Nilsson et al. 2004). Work with hardener-containing paints or plastics might induce asthma or allergic contact dermatitis. However, there is a very limited amount of scientific studies on exposures related to morbidity among seafarers.

The purpose of this survey was to get more information on current work exposures, the seafarers’ health status and their opinions on important occupational problems and safety.

## Materials and methods

 A total of 10,323 merchant seafarers (1535 women, 8788 men) were identified in the Swedish Maritime Registry (SR). Selection criteria for the study were a valid and personal e-mail address recorded in the SR (*N* = 4715 excluded, of whom 4617 had no e-mail address registered). The Web-based enquiry included different categories of questions: exposures, work-associated problems, health and work ability and safety climate. In total, the survey consisted of 170 questions (different sets of questions depending on work category) with an estimated required time of 30 min for completing the survey. A reference group representing stakeholders in the trade scrutinized the survey. The questionnaire is included as supplementary online information and accessible through the following link: http://maritimehealth.gu.se/english/research.

A total of 5608 seafarers were identified, all of whom received an invitation to answer the survey within a month’s time. Since we had no possibility of knowing the flag state of the seafarers’ work history, we specifically informed that the survey was directed to those having worked at least once since January 2010 on a Swedish flag state ship. After two reminders, 2220 (39%) had started the survey, of whom 1972 (35%) responded either completely (*N* = 1636) or almost (*N* = 336). Nine respondents were excluded as they were older than the general retirement age (66 years or older in 2010).

A validated short version of the NOSACQ50, called NOSACQ12, was used for qualitative measure of the safety climate, with “safety climate” here being described as the seafarers’ shared perceptions of their work leaders as well as the work group-related policies, procedures and practices in relation to safety (Kines 2011). Evaluation of the safety climate was divided into two different items to mirror (1) how the seafarers regarded their management’s/boss’ views on safety (“Management Safety Priority,” with questions, such as “Management is sure that all get the necessary information on safety that they need”), and (2) their co-workers views on safety (“General Security Climate,” with questions like “Those that work here try to find a solution when someone points out a safety problem”).

Unusual tiredness and its consequences (*fatigue*) were evaluated by the Modified Fatigue Impact Scale (MFIS). Isolated strain, or “*iso*-*strain,”* was evaluated according to the job-demand-control (JDC) model of Karasek and Theorell (Karasek et al. 1981, 1998; Theorell and Karasek 1996). For work ability, a validated single item question for a Work Ability Index (WAI) was used (Ahlstrom et al. 2010; Ilmarinen 2009).

Exposures were defined present if reported “daily or weekly,” and symptoms if reported “daily.” A work problem was considered present if reported “some,” “big” or “a very big.” A work problem is reported in percentage (%) of all respondents and not only of those exposed.

Statistics consisted of descriptive analyses for each work category. Differences in prevalence when comparing groups were calculated with Chi-square and *t* test, expressed in *p* values, with a 0.05 level of significance. For associations, prevalence ratios with 95% confidence intervals were calculated using the PROC PHREG procedure in SAS 9.4. All analyses were made using SAS© 9.4 and Windows Excel© 2010.

The study was based on informed consent and approved by the Regional Ethical Review Board of Gothenburg (Dnr 2013/811-12).

## Results

### Population characteristics

A total of 1963 eligible answers were collected, including 158 women and 1462 men (Table 1). Mean age of the respondents was 43 years (range 18–72). Most seafarers served on deck, followed by the engine room and service department, with only some (*N* = 12) not classifiable as to these three different work categories. Main vessel type of service was the RoPax/passenger ships (40%) and supply, service or research vessels (23%). Twelve percent were tank vessels. Half of the ships were on worldwide or European trades, while the rest were on sheltered and near costal trades. The vast majority had working schedules for between 2 weeks and 3 months, but some worked only day passes or were on board for 6 months up to a year. Eleven percent were smokers, and 33% ex-smokers, with mean years of smoking 29 and 15 years, respectively.

**Table 1:** Population characteristics as to work category and gender

	*N* (%)	Mean age (years)	Men *N* (%)	Women *N* (%)
Total	1963 (100%)	43	1462 (90%)	158 (10%)
Deck	1094 (61%)	42	893	89
Engine	551 (31%)	43	488	19
Service	124 (7%)	47	72	47
Other	12 (1%)	41	9	3

## Exposures

### Physical exposures

Exposure to noise was common for all work categories but especially in the engine room (engine room 89%; deck 52%; service 53%). Use of hearing protection was reported by 85% of noise exposed. Ergonomic strain, defined as having reported a weekly or daily exposure to any of the following subset of questions: “Which factor(s) do you experience [during your work on board]? (a) Strain on your arms, back or neck? (b) Uncomfortable work positions? (c) Heavy lifting?” was most commonly reported from the engine and service departments (88 and 85%, respectively) and to a lesser extent from the deck department (64%). An awkward work posture, strain on the neck, arm or back and heavy lifting were associated with an age below 30 years (*p* < 0.0001) and strain on the neck, arm or back and heavy lifting with female gender (*p* = 0.0001). In service, ergonomic strain was mainly due to strain on the neck, arm or back, while an awkward work posture was the most prevalent ergonomic exposure reported from the engine room.

Hand/arm vibrations (HAV) from handheld vibrating tools were reported by 24% of all seafarers irrespective of gender, type of ship or trade. HAV exposure was most common in the engine room (43% as to 16% in deck and service; *p* < 0.0001). It was also more common among ratings compared to officers (55% and 19%, respectively, *p* < 0.0001) and especially common among ratings in the engine room (75%) (Online resource Table 1).

### Chemical exposures

In the deck department, frequent (daily or weekly) chemical exposures consisted of exhausts (52%), different kinds of dust (39%) and oils on the skin (33%). A frequent use of solvent-based paints was reported from 16% and that of epoxy paints by 12%. In the engine department, frequent chemical exposures included oils on the skin (88%), exhausts (71%), oil mist (67%), solvent-based cleaning agents (65%) and soot (59%). Marine diesel oil and lubricant oils were the most common oils reported. In the service department, dust (51%) and non-solvent-based cleaning agents (39%) were the most commonly reported exposures. In the deck and engine departments, there were a number of exposures occurring less frequently (less than once a week), notably perhaps that of asbestos, thermosetting plastics, quite often a reported constitute in paints, and hydrazine (engine room) (Online Resource Figs. 1, 2). For all categories on board, 10% reported not having access to the safety protection needed in their work. This was more common among women compared to men (18, and 8%, respectively; *p* < 0.0001).

## Work environment problems and health

Anyone who had reported a rare, every week or daily occurring exposure received a subsequent question on how much of a work problem that specific exposure entailed (“no problem,” “some,” “big” or “very big”). The majority of those work problems were qualified as “some” (79%) and to a lesser extent as “big” (16%), or “very big” (5%).

**Fig. 1:**
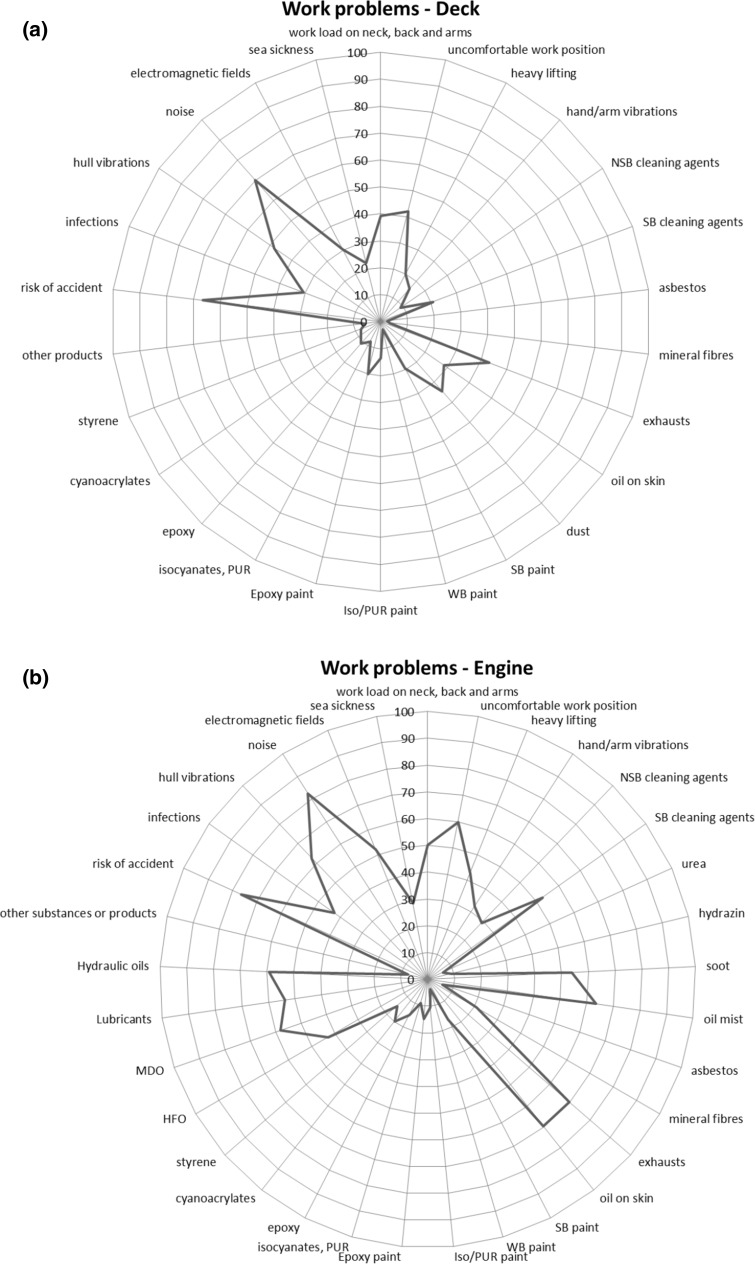

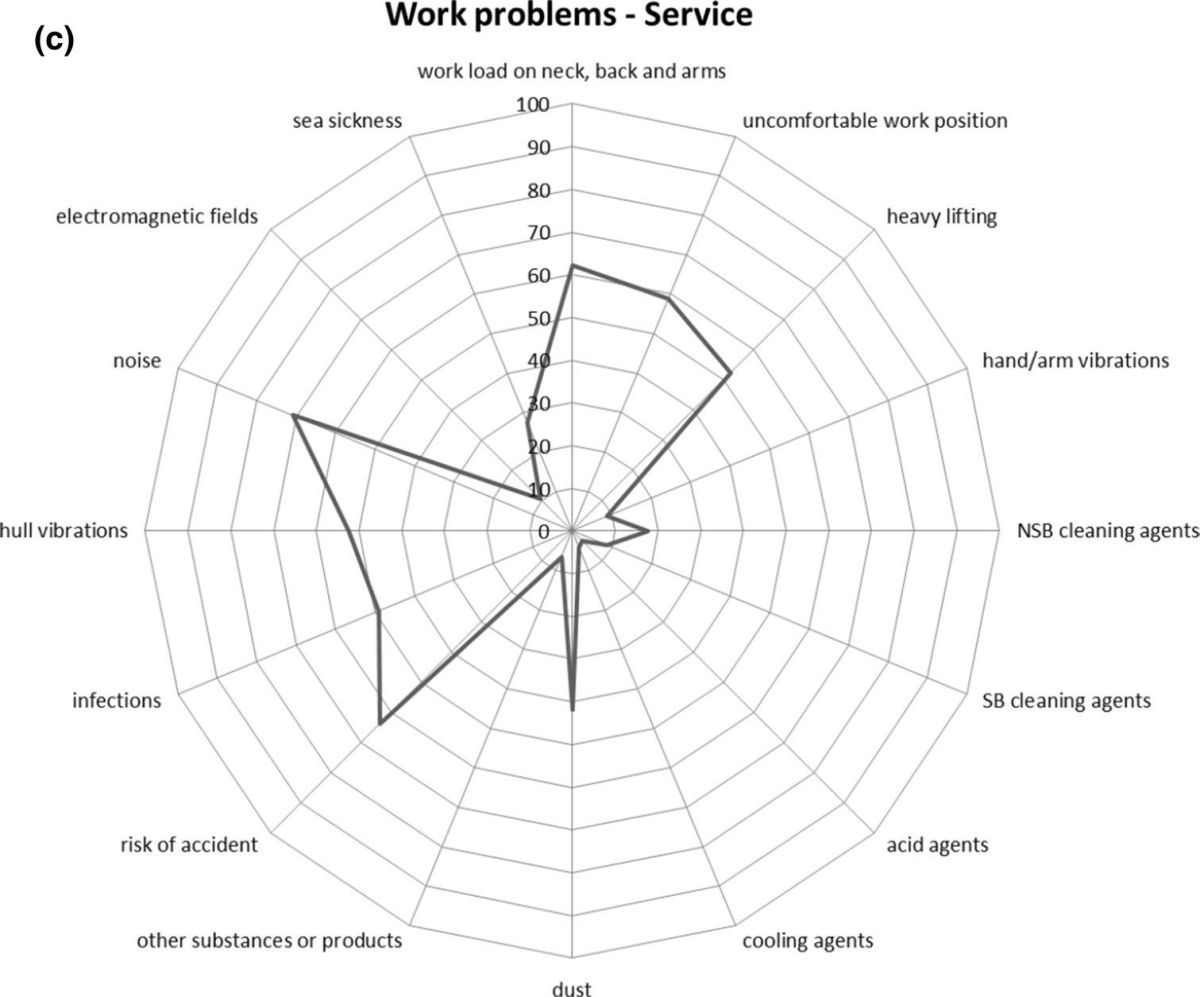
Work problems for each work category on board a merchant ship: deck (**a**), engine (**b**) and service (**c**). Percentage (%) for each exposure reported as a work problem

The most commonly reported work problems were noise (70% among deck respondents, 83% for engine and 71% for service) and risk of an accident (deck 67%, engine 77% and service 64%). Noise exposure was significantly associated with tinnitus or impaired hearing (PR 1.5; 95% CI 1.3–1.7). Other important work problems were vibrations from the hull (engine 63%), exhausts and skin contact with oils (engine both 70%), physical work load on neck, back and arms (service 62%) or risk of a contagious diseases (service 49%) (Fig. 1a–c). Symptoms from the lower airways, such as cough, were significantly associated with exposures to soot, dust and exhausts (Online resource Table 2).

Vibrations from the hull was primarily a work problem in trades farther out than the sheltered trade, in RoRo or car-carrying ships and tankers, and especially in icebreakers (72%). Experiencing hull vibrations as a work problem was more commonly reported in seafarers with a longer employment time (years) or with a higher position on board (Online Resource Table 3). Among those reporting vibrations from the hull as a work problem, pain or discomfort from the back, hips and knees were more common, as was unusual tiredness and sleep disturbances (Online resource Table 4).

It should be stressed, however, that the majority of seafarers reported a good or excellent health (77%) and a good or excellent work ability (deck 95%, engine 94%, service 85%). However, poor/moderate work ability and change of work tasks due to health reasons were both more frequently reported from women than from men (10%, and 6%, respectively; *p* = 0.04, and 26%, and 13%, respectively; *p* < 0.0001).

## Psychosocial factors and safety climate

Almost one-fourth of all crew members answered “Yes” to the question: “Have you at least once during the last 12 months felt exposed to offensive actions or harassment at your work place? For example—your actions or comments were ignored, you are not taken seriously, were ridiculed or patronized (y/*n*).” Although common among men (22%), offensive actions or harassment were twice as common in women (45%; PR 2.0; 95% CI 1.6–2.4, controlling for age). The majority of female engine room crew members reported on harassment or bullying, but they were few in total numbers (11/19; 58%).

The boss or the work leader was the most common culprit behind the offensive action or harassment (49% in total number of answers, where more than one answer would be possible), followed by a co-worker (34%), a passenger (9%) and any other category (8%). However, for women, the co-worker was the most common offender (46%).

Almost one-third (30%) of the service crew reported on iso-strain compared with 20% in deck and 11% in engine. Iso-strain was more common in women compared to men (27%, and 17%, respectively, *p* = 0.0027). Furthermore, it was associated with several symptoms, like headache and sleep disturbance with unusual tiredness (*p* < 0.0001). Iso-strain was also associated with having experienced harassment or offensive actions (*p* < 0.0001) (Online resource Table 5).

The safety climate was significantly higher in service crews compared with deck and engine crews (*p* = 0.02). It did not differ between the latter two. Lower safety estimates were associated with lower rank (non-managers vs. managers), having reported on unusual tiredness and being under 50 years of age. As to trade and type of ship, worldwide trade had the highest safety estimates, and passenger ships the lowest. No statistical significant differences were found as to sex (Online resource Table 6). There was a significant difference between the two items “Management Safety Priority” and “General Security Climate” (*p* < 0.0001), meaning that the respondents generally ranked the management’s/boss’ views on safety higher than the safety views of the co-workers. This difference still existed when stratified for having or not having a management position on board.

## Discussion

This descriptive study is, to our knowledge, unique in its comprehensiveness of work environment factors and the number of respondents, and we have obtained quantitative data on exposures, work problems and the psychosocial well-being of seafarers. As respondents were guaranteed anonymity and untraceability, we think they have been open about their opinions.

Noise, risk of accidents and whole-body vibrations were major work problems among seafarers within the Swedish merchant fleet. In our study, noise was a work problem especially in the engine room, and a higher prevalence of impaired hearing or tinnitus was found among engine room crew members. High levels of noise in the engine room are a well-known problem (Ivergård et al. 1978; Kaerlev et al. 2008; Svendsen and Borresen 1999; Wagner et al. 2008), but there are no available studies on the sources of noise for the deck and service departments. Rising epidemiological evidence suggests that noise increases the risk of high blood pressure, sleeping disturbance and fatigue, obesity and diabetes mellitus, all of which have been shown to be more common in seafarers (Bloor 2000; Borch et al. 2012; Brandt et al. 1994; Elo 1985; Jensen 1996). Furthermore, noise may be an all-time present exposure on a ship and a continuous stressor to the seafarer.

Whole-body vibrations (WBV) have been linked to a higher risk of back pain, but studies have also found associations with sleep disturbances, e.g., people living next to the railway, and gestational hazards, like preterm birth or being small for gestational age (Burstrom et al. 2015; Seidel 1993; Waddington et al. 2015). Today, there is a lack of knowledge regarding levels of WBV and health effects in the merchant fleet (Jensen and Jepsen 2014). In our study, pain or discomfort in the back, hips and knees as well as sleep disturbances and unusual tiredness were more commonly reported if exposed to whole-body vibrations (WBV).

Ergonomic strain represented an important exposure as well as a work problem in all work categories. In the engine room, ergonomic strain has been linked to the ship design, but knowledge is especially lacking for the other departments (Lundh 2010; Lundh et al. 2011).

Exposure to carcinogens from different oils might occur through inhaled oil mist and dermal contact with oils, the latter supposedly being the most relevant in an every day setting and especially for work in the engine room (Forsell et al. 2007; Nordlinder and Nilsson 1999a, b, 2001). In our study, 88% of the engine room crew had dermal exposure to oils at least once a week, the majority reporting an every day exposure (62%). It has been postulated that oil dermal exposure is one possible cause for the higher cancer incidence in engine room personal, making this exposure very relevant for intervention measures (Forsell et al. 2007). It is worth noting, that dermal exposure to oils was not only reported from the engine: One-third of the deck crew also reported such an exposure.

The safety climate was estimated higher by seafarers with a management function on the ship. It is known from other occupations that having a management role increases the likelihood of an overestimation of the safety climate, being more responsible for the safety organization itself (Torner et al. 2011). However, it should be stressed that all safety estimates in our study were considerably higher when compared to land-based occupations (*data from NOSACQ research group*). High safety values have been found in other studies on seafarers (Jensen et al. 2005). This is perhaps not so surprising in view of the vulnerability when working on the seas.

Although few in number of respondents, women seafarers come forward in this study as a specific subgroup of conflicts between gender and occupational risks. Women reported more than men on lack of a proper safety equipment, more of ergonomic strain and psychosocial factors in terms of iso-strain and harassments. Some of the occupational exposures reported might also be relevant in risk assessment of a pregnancy, like exposures to solvents, noise and whole-body vibrations.

As to the selection of the study population, 45% of seafarers in the Swedish Maritime Registry did not have an e-mail address. The reasons for not having an e-mail address in the Registry are not known—the Authority does not ask for it, but it is given as an option to the seafarer in contact matters. There were no major differences as to sex or age between those with and those without an e-mail address in the Seafarers’ Registry. Another significant limitation of the study is that the response rate was low (35%). Since we had no access to data other than the seafarers’ e-mail addresses, we do not know why so many left the questionnaire unanswered. In fact, non-responders might just have rightfully decided they were not targeted by the survey. Although precautions were taken not to classify our mail as spam by the respondent’s server, this possibility also cannot be excluded. We could see though that the study population was a representative sample compared with the total number of seafarers in the Seafarers’ Registry in terms of gender and age. A sensitivity analysis had only minor impact on differences between groups and associations between exposures and symptoms.

## Conclusions

Noise, risk of an accident and whole-body vibrations are important work problems for Swedish seafarers in the merchant trade. Health problems were more common if exposed to noise or vibrations, indicating the need to reduce these exposures. Chemical exposures for exhausts, soot, oil on the skin and oil mist were commonly reported, but also different kinds of plastics and paints. Social factors and the nature of working at sea might explain why offensive actions or harassment were so common, but the causes and mechanisms behind these results need further clarification.

## Electronic supplementary material

Below is the link to the electronic supplementary material.

Supplementary material 1 (DOCX 72 kb)
