# Supplementary material for: Work environment and safety climate in the Swedish merchant fleet
Source: Int Arch Occup Environ Health. 2016 Nov 4;90(2):161–8. doi: 10.1007/s00420-016-1180-0 (PMC5263194; doi:10.1007/s00420-016-1180-0)
Supplement: Supplementary file 1 — Supplementary material 1 (DOCX 72 kb) [file 420_2016_1180_MOESM1_ESM.docx]

**Online Resources**

**Table 1**  Exposure to hand/arm vibrations daily or weekly for seafarers

|  | | **Number** | **Percentage (%)** |
| --- | --- | --- | --- |
|  | Total | 1681 | 24 |
| **Category** | Deck | 1036 | 16 |
|  | Engine | 531 | 43 |
|  | Service | 114 | 16 |
| **Age** | 18-30 yrs | 335 | 36 |
|  | 31-40 yrs | 421 | 24 |
|  | 41-50 yrs | 338 | 20 |
|  | >50 yrs | 471 | 17 |
| **Position** | Officer – deck | 789 | 7 |
|  | Rating – deck | 173 | 50 |
|  | Officer - engine | 456 | 38 |
|  | Rating - engine | 48 | 75 |
| **Type of ship** | Ro/Ro, or similar | 240 | 25 |
|  | Ro/pax, Passenger ship | 642 | 25 |
|  | Container, Cargo, Bulk | 31 | 35 |
|  | Tank | 246 | 15 |
|  | Supply, Service or Research vessels | 379 | 26 |
| **Trade** | Sheltered | 223 | 12 |
|  | Near coastal | 573 | 30 |
|  | European | 489 | 24 |
|  | Worldwide | 370 | 22 |
| **Years as a seafarer** | 0-5 yrs | 370 | 36 |
|  | 6-15 yrs | 533 | 25 |
|  | >15 yrs | 762 | 18 |


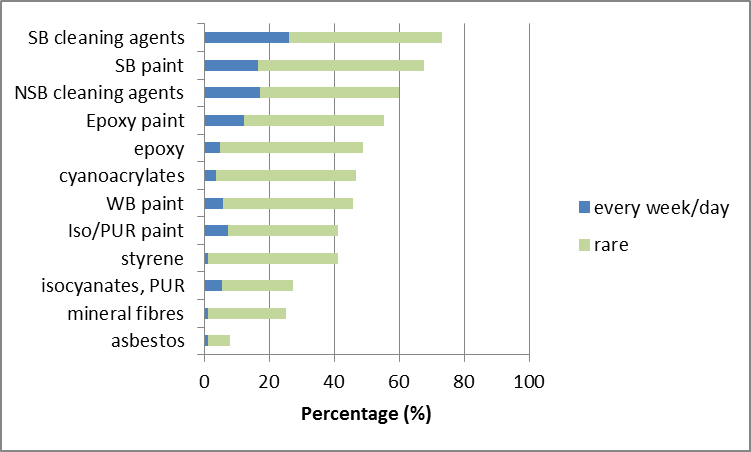


**Fig 1**  Selection of potentially hazardous chemicals with rare, every week or daily exposures on deck. SB = solvent based; NSB = non solvent based; WB = water based


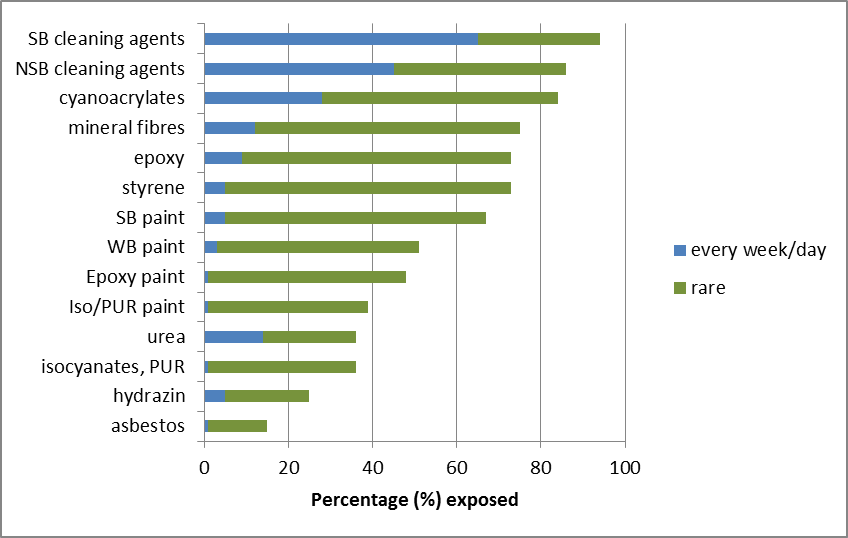


**Fig 2**  Selection of potentially hazardous chemicals showing rare, exposures in the engine room. SB = solvent based; NSB = non solvent based; WB = water based

**Table 2** Associations between air way exposures and symptoms from the lower airways. Statistical significant associations were found for lower airway symptoms and exposures to soot*, exhausts† or dust^

|  | Lower airway symptoms | | | | | |  | | |
| --- | --- | --- | --- | --- | --- | --- | --- | --- | --- |
|  | Exposed | | | Non-exposed | | |  |  |  |
|  | Obs  (N) | Cases  (N) | Percentage (%) | Obs  (N) | Cases  (N) | Percentage  (%) | PR | 95% CI | |
| Soot* | 300 | 28 | 9.3 | 202 | 8 | 4.0 | 2.4 | 1.1 | 5.1 |
| Oil mist* | 340 | 28 | 8.2 | 165 | 8 | 4.9 | 1.7 | 0.8 | 3.6 |
| Exhausts† | 854 | 54 | 6.3 | 619 | 4 | 32.0 | 1.6 | 1.0 | 2.5 |
| Dust^ | 432 | 32 | 7.4 | 655 | 24 | 3.7 | 2.0 | 1.2 | 3.4 |

*) engine; †) deck & engine; ^) deck & service

**Table 3**  Number (N) of seafarers for each subgroup and respective percentage (%), that reported vibrations from the hull as a work problem

|  | | **N seafarers** | **Percentage (%)** |
| --- | --- | --- | --- |
| **Trade** | Sheltered | 268 | 40 |
|  | Near coastal | 624 | 52 |
|  | European | 523 | 59 |
|  | Worldwide | 393 | 53 |
| **Position** | Officer - deck | 810 | 54 |
|  | Rating - deck | 176 | 41 |
|  | Officer - engine | 466 | 67 |
|  | Rating - engine | 48 | 52 |
| **Years as a seafarer** | 0-5 yrs | 489 | 33 |
|  | 6-15 yrs | 582 | 53 |
|  | >15 yrs | 824 | 58 |
| **Type of ship** | Ro/Ro, or similar | 245 | 64 |
|  | Ro/pax, Pass. | 695 | 51 |
|  | Container, Cargo, Bulk | 34 | 50 |
|  | Tank | 252 | 62 |
|  | Supply, Service or Research vessels | 411 | 53 |
|  | Other | 133 | 36 |
|  | Ice breakers | 46 | 72 |

**Table 4**  Presence of symptoms if exposed to hand/arm vibrations (HAV) or not (non-HAV), and if having reported vibrations from the hull as a work problem (whole body vibrations, WBV) or not (non-WBV). Prevalence ratios and 95% confidence intervals

|  | N | (%) | N | (%) |  |  |  |
| --- | --- | --- | --- | --- | --- | --- | --- |
|  | HAV |  | non-HAV |  | PR | 95% CI | |
| **White fingers** | 370 | 21 | 1199 | 15 | 1.43 | 1.12 | 1.82 |
| **Numbness/pricking** | 370 | 19 | 1195 | 10 | 1.91 | 1.45 | 2.50 |
|  | | | | | | | |
|  | WBV |  | non-WBV |  | PR | 95% CI | |
| **Back – pain** | 295 | 41 | 1312 | 32 | 1.27 | 1.08 | 1.48 |
| **Hips - pain** | 292 | 20 | 1303 | 9 | 2.19 | 1.65 | 2.92 |
| **Knees - pain** | 295 | 39 | 1305 | 23 | 1.70 | 1.43 | 2.03 |
| **Sleep disturbance** | 302 | 52 | 1332 | 42 | 1.25 | 1.10 | 1.41 |
| **Unusual tiredness** | 302 | 38 | 1330 | 25 | 1.52 | 1.28 | 1.81 |

**Table 5** Conception of risk of an accident as a work problem, having experienced offensive (off.) actions or harassments at work, and perception of health and symptoms divided by experiencing strain at work without social support (Iso-strain) or not (No iso-strain). Number (N) of answers with prevalence ratios (PR) and 95% confidence intervals (CI), controlling for sex

|  | Answers (total) | Iso-strain | No iso-strain |  | |
| --- | --- | --- | --- | --- | --- |
|  | N | N | N | PR | CI |
| Risk of an accident | 1583 | 85 (31%) | 191 (15%) | 2.01 | 1.60-2.51 |
| Off. Action/harassment | 1589 | 128 (46%) | 261 (20%) | 2.59 | 2.08-3.21 |
| Good or excellent health | 1585 | 175 (63%) | 1050 (80%) | 0.50 | 0.41-0.63 |
| Headache | 1576 | 19 (7%) | 32 (2%) | 2.19 | 1.51-3.18 |
| Tiredness | 1572 | 42 (15%) | 52 (4%) | 2.68 | 2.06-3.48 |
| Sleep disorder | 1576 | 44 (16%) | 63 (5%) | 2.37 | 1.82-3.10 |
| Anxiety | 1574 | 21 (8%) | 33 (3%) | 2.05 | 1.41-2.98 |
| Unusual tiredness | 1574 | 126 (45%) | 308 (24%) | 2.18 | 1.77-2.70 |
| Poor or moderate work ability | 1565 | 32 (12%) | 61 (5%) | 1.99 | 1.46-2.71 |
|  |  |  |  |  |  |
| *Women* | *1542* | *39 (14%)* | *108 (9%)* | *1.60^* | *1.19-2.14* |

^) not controlling for gender

**Table 6**  Point estimates of safety climate in seafarers sailing under a Swedish flag, illustrating the seafarers view on their leaders views on safety (Management Safety Priority), and their own views (General Security Climate).

|  | |  | Management Safety Priority | | General Security Climate | |
| --- | --- | --- | --- | --- | --- | --- |
|  |  | N | Mean | *p* | Mean | *p* |
| Work category | deck* | 938 | 3.36 |  | 3.32 |  |
|  | engine | 480 | 3.36 | ns | 3.32 | ns |
|  | service | 116 | 3.51 | 0.0187 | 3.44 | 0.0165 |
| Sex | men* | 1374 | 3.38 |  | 3.33 |  |
|  | women | 147 | 3.30 | ns | 3.26 | 0.0803 |
| Management | Manager* | 1157 | 3.40 |  | 3.35 |  |
|  | Non manager | 374 | 3.29 | 0.0049 | 3.24 | 0.0002 |
| Unusual tiredness | no* | 1114 | 3.42 |  | 3.38 |  |
|  | yes | 426 | 3.22 | <0.0001 | 3.19 | <0.0001 |
| Age | <30 | 327 | 3.29 | <0.0001 | 3.21 | <0.0001 |
|  | 31-40 | 402 | 3.30 | <0.0001 | 3.26 | <0.0001 |
|  | 41-50 | 348 | 3.37 | 0.0097 | 3.36 | 0.0115 |
|  | >50* | 459 | 3.49 |  | 3.45 |  |
| Trade | Sheltered* | 220 | 3.24 |  | 3.29 |  |
|  | Near coastal | 552 | 3.31 | ns | 3.26 | ns |
|  | European | 437 | 3.37 | 0.0169 | 3.34 | ns |
|  | Worldwide | 346 | 3.56 | <0.0001 | 3.45 | <0.0001 |
| Type of ship | Ro/Ro. or similar* | 230 | 3.46 |  | 3.40 |  |
|  | Ro/pax. Pass. | 604 | 3.30 | 0.0014 | 3.28 | 0.0007 |
|  | Container. Cargo. Bulk | 24 | 3.48 | ns | 3.36 | ns |
|  | Tank | 239 | 3.38 | ns | 3.35 | ns |
|  | Supply. Service or Research vessels | 353 | 3.43 | ns | 3.38 | ns |
|  | other | 83 | 3.34 | ns | 3.27 | 0.0287 |

*)Referent; ns=non significant
